# Artemisinin exerts antidepressant-like effects via activation of AKT and ERK signaling pathways

**DOI:** 10.3389/fphar.2025.1642167

**Published:** 2025-10-17

**Authors:** Ruohong Lin, Zhiwei Zhou, Yizhou Jiang, Song Liu, Jinfeng Xie, Haitao Wang, Henning Ulrich, Wenhua Zheng

**Affiliations:** ^1^ Pharmaceutical Science, Faculty of Health Sciences, University of Macau, Taipa, Macao SAR, China; ^2^ Research and Development Department, Zhuhai Tengbai Pharmaceutical Co., Ltd., Zhuhai, China; ^3^ International Center for Aging and Cancer, Hainan Academy of Medical Sciences, Hainan Medical University, Haikou, China; ^4^ NMPA Key Laboratory for Research and Evaluation of Drug Metabolism and Guangdong Provincial Key Laboratory of New Drug Screening, School of Pharmaceutical Sciences, Southern Medical University, Guangzhou, China; ^5^ Guangdong-Hong Kong-Macao Joint Laboratory for New Drug Screening, School of Pharmaceutical Sciences, Southern Medical University, Guangzhou, China; ^6^ Departamento de Bioquimica, Instituto de Quimica Universidade de Sao Paulo, São Paulo, Brazil; ^7^ Guangdong-Hong Kong-Macao Joint Laboratory for New Drug Screening, University of Macau, Taipa, Macao SAR, China

**Keywords:** artemisinin, depression, corticosterone, chronic unpredictable mild stress, oxidative stress, Akt/Erk signaling

## Abstract

**Aims:**

Depression is a leading cause of disability worldwide, with current treatments often limited by efficacy and side effects. Artemisinin (ART), a natural compound with known anti-inflammatory and neuroprotective properties, has not been extensively studied for its potential antidepressant effects. This study aimed to elucidate the neuroprotective mechanisms of artemisinin against corticosterone (CORT)-induced toxicity in PC12 cells model, and to assess its antidepressant-like behavioral effects in a chronic unpredictable mild stress (CUMS) mouse model.

**Methods:**

*In vitro*, PC12 cells and primary hippocampal neurons were treated with CORT and artemisinin to assess cell viability, oxidative stress, mitochondrial function, and apoptosis. Pharmacological inhibition and CRISPR/Cas9 gene editing were used to explore the roles of AKT and ERK signaling pathways. *In vivo*, CUMS-induced depression-like behaviors in mice were evaluated using sucrose preference, tail suspension, and forced swim tests. Western blotting and immunohistochemistry studies were performed to analyze molecular mechanisms.

**Results:**

Artemisinin attenuated CORT-induced cytotoxicity, oxidative stress, mitochondrial dysfunction, and apoptosis in PC12 cells and hippocampal neurons. These effects were mediated through the activation of AKT and ERK pathways. In CUMS mice, artemisinin improved depression-like behaviors, upregulated the AKT/GSK/NRF2/HO1 and BDNF/TrkB/ERK/CREB pathways, modulated astrocyte activity, and promoted neurogenesis in the hippocampus.

**Conclusion:**

Artemisinin exerts significant neuroprotective and antidepressant-like effects through multiple molecular and cellular mechanisms, highlighting its potential as a novel therapeutic agent for depression.

## 1 Introduction

Depression is a major mental disorder, often associated with significant symptom severity such as cognitive deficits, persistent negative mood, and physiological impairments (Brzezicka, 2013; Elderkin-Thompson et al., 2007; Levin et al., 2007; Nutt et al., 2008). These symptoms can severely limit patients’ ability to carry out major life activities. Moreover, according to the World Health Organization (WHO), major depression can lead to more than 700,000 suicides per year globally. As of 2017, over 300 million people around the world suffered from depression (WHO, 2017), and it is predicted that by 2030, major depressive disorder will become the leading cause of disability worldwide (Mathers and Loncar, 2006). Previous research has evidenced an increasing trend in the incidence of depression (Mojtabai et al., 2016; Weinberger et al., 2018). Currently, antidepressant medication is a primary treatment modality for depression. Various types of antidepressants, including monoamine oxidase inhibitors (MAOIs), tricyclic antidepressants (TCAs), and selective serotonin reuptake inhibitors (SSRIs), are available for clinical treatment. While newer agents such as SSRIs demonstrate improved safety profiles compared to older TCAs, it is crucial to acknowledge that all pharmacological agents carry inherent risks of adverse effects, common side effects associated with antidepressants include gastrointestinal disturbances, sexual dysfunction, and withdrawal symptoms (Carvalho et al., 2016; Kennedy, 2006; Rush et al., 2006; Strawn et al., 2023). Furthermore, approximately 30% of major depressive disorder patients fail to respond adequately to existing antidepressant therapies (Al-Harbi, 2012). These limitations, suboptimal efficacy and the unavoidable potential for adverse reactions, collectively underscore the pressing need to develop novel, effective, and safer antidepressants.

Artemisinin (ART), extracted from the plant Artemisia annua, has been used as a remedy by traditional herbal medicine practitioners in China for over 2000 years (Maude et al., 2010). Since the late 1990s, artemisinin compounds have served as frontline antimalarial drugs (Miller and Su, 2011). Due to its ability to pass the blood-brain barrier (BBB) and its low side effect profile in clinical use, artemisinin presents favorable advantages for treating other diseases. Beyond its antimalarial effects, artemisinin exhibits a wide range of pharmacological properties, including antiviral, anti-inflammatory, antioxidant, and antitumor activities (Efferth et al., 2008; Firestone and Sundar, 2009; Ho et al., 2014; Li et al., 2012; Zhou et al., 2025). Importantly, accumulating evidence indicates that artemisinin could also be a drug candidate for treating neurodegenerative diseases such as Alzheimer’s disease (AD) and Parkinson’s disease (PD). For instance, *in vivo* and *in vitro* experiments have shown that artemisinin has protective effects on Alzheimer’s disease pathology by activating the ERK pathway (Zeng et al., 2017; Zhao X. et al., 2020) or suppressing inflammasome activation (Shi et al., 2013). Artemisinin also shows protective effects in 1-methyl-4-phenylpyridinium (MPP+)-induced cellular models of Parkinson’s disease (Yan et al., 2021). Additionally, it can protect various neuronal cells from oxidative stress damage (Deng et al., 2024; Lin et al., 2018; Zhao et al., 2019a; Zheng et al., 2016). Critically, recent studies specifically support its antidepressant potential: Artesunate (an artemisinin derivative) prevented H_2_O_2_-induced oxidative damage in PC12 cells and attenuated LPS-triggered depression-like behaviors in mice via suppressing neuroinflammation and oxidative stress (Huang et al., 2023). Specifically, a novel dihydroartemisinin (an artemisinin derivative)-GABA conjugate (5b) demonstrated potent protective effects against corticosterone-induced impairments in PC12 cells (a model relevant to depression) (He et al., 2021). Given these facts, artemisinin and its derivatives are potential candidates for antidepressant drugs. However, there is little report on the protective effects of artemisinin itself on animal and cell models of depression.

Despite the high prevalence of depression, its underlying mechanisms remain unclear. There is no single cause of the pathogenesis of depression; environmental stress, brain biochemical alterations, and genetic vulnerability all contribute (Ruiz et al., 2018). Studies have suggested that excessive stress plays an important role in the development of major depressive episodes (Pariante, 2003; Wang, 2005). Stressful events activate the hypothalamic-pituitary-adrenal (HPA) axis, leading to hyperactivity and the generation of excessive concentrations of circulating glucocorticoids. Increasing evidence indicates that the hippocampus is sensitive to glucocorticoids, exhibiting both structural and functional changes (Holmes and Wellman, 2009; Lupien et al., 1998; Sapolsky, 2000). Clinical studies also show that the hippocampal volume of patients with depression is smaller than that of healthy individuals (Den Heijer et al., 2011; Nollet et al., 2013; Willner, 1997).

Although glucocorticoids help the body deal with stress, the abundance of glucocorticoid receptors in hippocampal tissue means that high levels of corticosterone (a type of glucocorticoid) can cause damage to nerve cells and hippocampal dysfunction, eventually inducing depression-like behaviors in mice. PC12 cells, derived from a pheochromocytoma of the rat adrenal medulla, possess typical features of brain neurons and contain abundant glucocorticoid receptors. Thus, corticosterone (CORT)-induced damage to PC12 cells has been widely used as a tool for *in vitro* anti-depression pharmacological research (Wang et al., 2013; Zeng et al., 2016).

Given that socio-environmental chronic stressors are involved in the development of depression, the chronic unpredictable mild stress (CUMS) model is extensively used *in vivo* to elucidate the biological mechanisms of depression and screen antidepressant drugs. This model can elicit depression-like symptoms such as anhedonia, as evidenced by decreased sucrose preference, and these abnormalities can be reversed by antidepressant administration. Accordingly, the PC12 cellular model combined with the CUMS animal model was applied to assess the potential antidepressant effects of artemisinin in this study.

The aim of the present study was to examine antidepressant-like activity of artemisinin, by means of corticosterone-induced PC12 cell model and CUMS mice model, as well as its underlying mechanism. These findings could suggest that artemisinin holds promise as a potential drug for the prevention and treatment of depression.

## 2 Materials and methods

### 2.1 Materials

Analytical-grade artemisinin, CORT, and Fluoxetine were obtained from Meilunbio (Dalian, China). Dulbecco’s modified Eagle’s medium (DMEM), fetal bovine serum (FBS), and 0.25% trypsin were from GIBCO (USA). DMSO and penicillin/streptomycin were sourced from Sigma-Aldrich (USA). Poly-D-lysine, MTT, Lipofectamine 3000, and BCA Protein Assay Kit were from Thermo Fisher Scientific (USA). Annexin V: FITC Apoptosis Detection Kit was from BD Biosciences (USA). SDS-PAGE gels (4%–20%) were purchased from Genscript Biology (China). Primary antibodies for GAPDH, phospho-AKT1, phospho-ERK1/2, phospho-CREB, phospho-TrkB, phospho-GSK3β, NeuN, and HRP-conjugated secondary antibodies were from CST (USA). BDNF antibody was from Abcam (UK), while GFAP, NRF2, and HO1 antibodies were from Signalway Antibody (USA). MEK inhibitor PD98059 and PI3K inhibitor LY294002 were from Calbiochem (USA). Lactate dehydrogenase (LDH) cytotoxicity, mitochondrial membrane potential (MMP, △ψm; JC-1) and reactive oxygen species (ROS) detection kits were from Beyotime Biotechnology (China).

### 2.2 Cell culture and treatment

PC12 cells were provided by Dr. Gordon Guroff (NIH, USA) and cultured in DMEM with 10% FBS and penicillin/streptomycin at 37 °C in 5% CO2. Cells were sub-cultured 2–3 times per week at a 1:5 split. Primary neurons were obtained from one-day-old C57BL/6 mice as per Zhao and colleagues (Zhao et al., 2019b). Artemisinin and CORT were dissolved in DMSO (≤0.1% final concentration). Experimental groups included: (1) Control: serum-free medium, (2) CORT group: 200 μM CORT, and (3) CORT + Artemisinin: 200 μM CORT with 3.125–100 μM artemisinin for 48 h.

### 2.3 MTT assay

Cell viability was assessed using the MTT assay. Cells (6–8 × 10^3^/well) were treated with artemisinin and CORT for 48 h, followed by incubation with 0.5 mg/mL MTT for 3–4 h. Formazan crystals were dissolved in 150 μL DMSO, and absorbance was measured at 490 nm using an Infinite M200 PRO microplate reader (Tecan, Switzerland). Cell viability was expressed as a percentage of control.

### 2.4 LDH cytotoxicity assay

LDH release was measured using a commercial kit (Beyotime). Cells (6–8 × 10^3^/well) were treated as described, and fluorescence intensity was measured at 560/590 nm using an Infinite M200 PRO microplate reader. LDH release was normalized to the control group.

### 2.5 ROS detection

ROS levels were detected using DCFH-DA (Beyotime). Cells were incubated with 10 μM DCFH-DA in DMEM at 37 °C for 30 min, washed, and fluorescence was observed using an EVOS FL Imaging System. ROS levels were semi-quantified using ImageJ.

### 2.6 Mitochondrial membrane potential (MMP) assay

MMP (∆ψm) was assessed using the JC-1 kit (Beyotime). Cells (1 × 10^4^ cells/cm^2^) were treated for 48 h, stained with JC-1 (10 μg/mL) at 37 °C for 20 min, washed, and fluorescence was analyzed using a fluorescence microscope. The red/green fluorescence ratio was normalized to the control group.

### 2.7 Apoptosis assay

Apoptosis was measured using an Annexin V-FITC/PI apoptosis detection kit (BD Biosciences, San Diego, CA, USA) according to the manufacturer’s instructions. Cells were treated for 48 h, then 1 × 10^5^ cells were collected and stained with Annexin V-FITC and PI. Based on the assay principle, intact cells were double-negative for Annexin V and PI; early apoptotic cells were identified as Annexin V-positive/PI-negative, while late apoptotic or necrotic cells were positive for both Annexin V and PI. To quantify the apoptotic cells, CellQuest™ Pro Software (BD Biosciences, San Diego, CA) was utilized, and the apoptosis rate was determined as the percentage of cells positive for Annexin V.

### 2.8 CRISPR/Cas9 gene editing

The gene editing was carried out as previously described (Ran et al., 2013). Rat AKT1 was targeted using sgRNA sequences rAKT1-gRNA-F1: CAC​CGA​GGT​GCC​ATC​ATT​CTT​GAG​G and rAKT1-gRNA-R1: AAA​CCC​TCA​AGA​ATG​ATG​GCA​CCT​C. The sgRNA was cloned into PX459 V2.0 and transfected into PC12 cells using lipofectamine 3000. Cells were screened with puromycin, and AKT1 expression was verified by Western blot.

### 2.9 Western blot analysis

Protein lysates were prepared from the mouse hippocampus or PC12 cells using RIPA buffer with protease and phosphatase inhibitors. Protein concentrations were determined using the BCA assay. Samples (20 μg) were separated via 4%–20% SDS-PAGE, transferred to PVDF membranes, blocked with 5% BSA, and incubated overnight with primary antibodies (1:500–2000) at 4 °C. Membranes were incubated with HRP-conjugated secondary antibodies (1:4000) for 2 h, and bands were detected using ECL. Protein expression was normalized to GAPDH. The intensity of the bands was semi-quantified by using ImageJ software.

### 2.10 Animal studies and drug administration

Male C57BL/6 mice (n = 48, 6–8 weeks, 18–21 g) were housed under standard conditions (25 °C, 12 h light/dark cycle) with food and water *ad libitum*. All procedures followed the University of Macau Animal Ethics Committee guidelines. Mice were divided into six groups: Control, CUMS, artemisinin (0.3, 1 and 3 mg/kg), and fluoxetine (10 mg/kg) which is a selective serotonin reuptake inhibitor (SSRI) and well-established antidepressant used as a positive control in this study (Dulawa et al., 2004; Levy et al., 2019). Drugs were administered via intraperitoneal injection 30 min after stress daily for 4 weeks. Mice were sacrificed post-behavioral tests.

### 2.11 Chronic unpredictable mild stress (CUMS) protocol

The CUMS procedures were performed according to previous reports (Xu et al., 2020; Zhao Y.-N. et al., 2020; Zhu et al., 2020), with some modifications. CUMS was applied for 5 weeks with random exposure to stressors, with each stressors applied 3–4 times per week: (1) overnight flash illumination, (2) 8-h ultrasonic noise, (3) 12-h wet bedding, (4) 12-h water deprivation, (5) 24-h food deprivation, (6) 4-h cage tilt, (7) 1-min tail nip, (8) 5-min cold swim (4 °C), (9) 2-h physical restraint, (10) 8-h exposure to a pungent odor, (11) overnight light exposure. After 5 weeks of CUMS exposure, mice were subjected to different behavioral tests such as sucrose preference test (SPT), Tail suspension test (TST) and forced swim test (FST).

### 2.12 Behavioral tests

SPT: The test was carried out based on previously established methods, with a few alterations (Liu et al., 2015). Briefly, mice were trained to consume 1% sucrose solution, followed by 12 h water deprivation. Sucrose preference was calculated as (sucrose intake/total liquid intake) × 100%. TST: This test was performed based on the previous description (Steru et al., 1985), with slight modification. Briefly, mice were suspended by the tail (60 cm above ground) for 5 min. Immobility duration was recorded. FST: The test was conducted according to the method of Porsolt, with some modifications (Porsolt et al., 1977). Briefly, mice were placed in water-filled cylinders (25 °C ± 2 °C) for 5 min. Immobility was recorded during the last 4 min.

### 2.13 Immunohistochemistry and immunofluorescence

Mice were perfused with PBS, and brains were fixed in paraformaldehyde. Sections (5 μm) were dewaxed, rehydrated, blocked, and incubated with primary antibodies (1:200) overnight at 4 °C. For DAB staining, sections were treated with secondary antibodies, followed by color development. For immunofluorescence, sections were incubated with Alexa Fluor 488-conjugated secondary antibodies (1:500) and counterstained with DAPI. Images were acquired using an EVOS FL Imaging System or Carl Zeiss Axio Observer.

### 2.14 Statistical analysis

*In vitro experiments were performed in at least three independent replicates. In vivo data are based on a sample size of 8 animals per group*. The data analysis was conducted by GraphPad Prism 8.0 statistical software (GraphPad software, Inc., SanDiego, CA, USA) and presented as mean ± SD. Statistical significance was determined using unpaired t-test for comparisons between two groups, and one-way ANOVA followed by *post hoc* Tukey’s test for multiple group comparisons. Statistical significance was defined as p < 0.05, P < 0.01 or P < 0.001.

## 3 Results

### 3.1 Artemisinin (ART) attenuated the decrease in cell viability caused by CORT in PC12 cells

**FIGURE 1:**
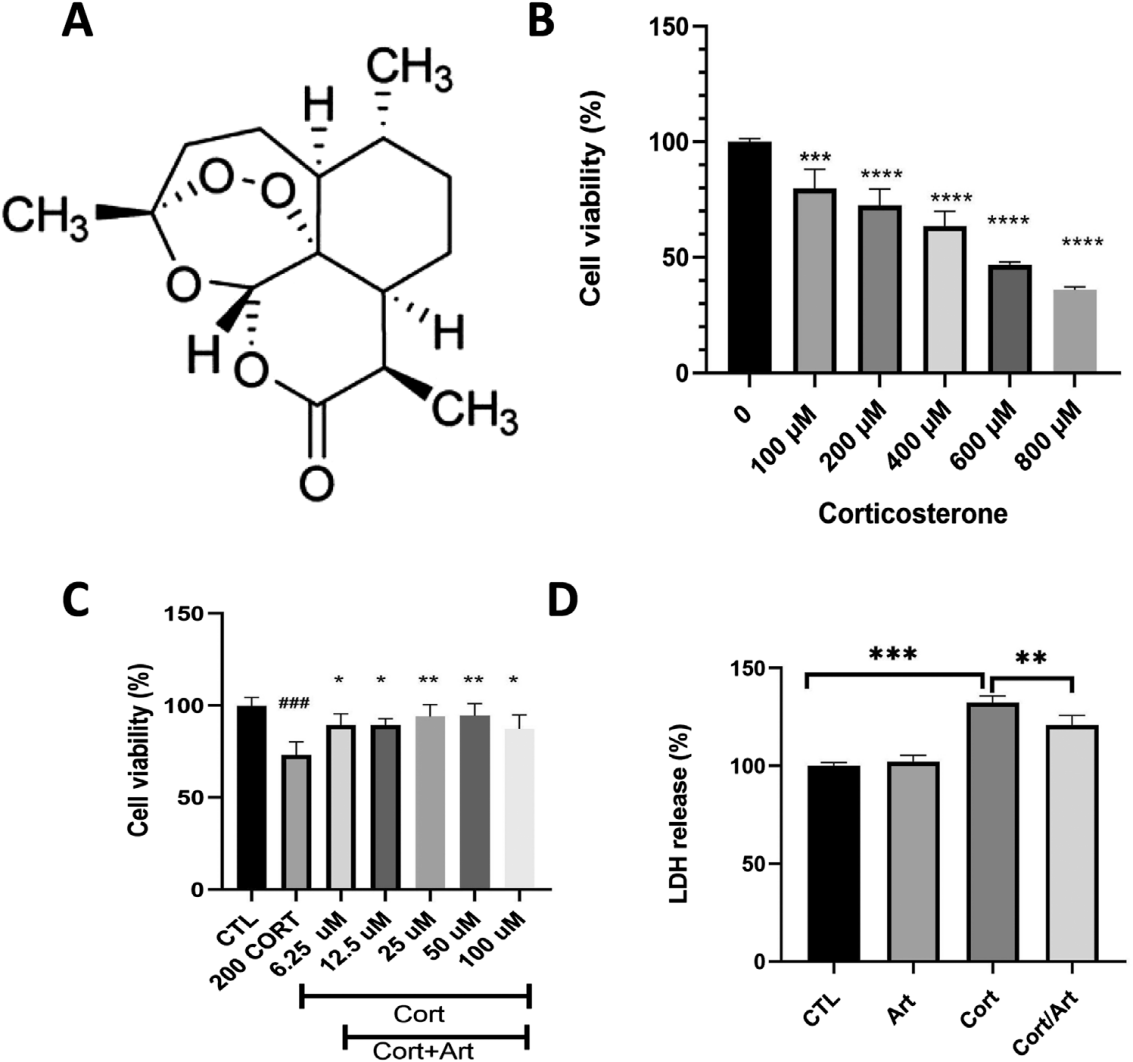
Artemisinin mitigates the reduction in cell viability caused by CORT in PC12 cells. **(A)** The structure of artemisinin. **(B)** The cytotoxicity of CORT on PC12 cells. Cells were treated with CORT (100–800 μM) for 48 h, and cell viability was measured using the MTT assay. **(C)** Artemisinin mitigates the reduction in cell viability induced by CORT in PC12 cells. Cells were co-treated with artemisinin and corticosterone at indicated concentrations for 48 h, and cell viability was measured using the MTT assay. **(D)** Cells were co-treated with artemisinin and CORT for 48 h, and cytotoxicity was measured using the LDH assay. Data represent means ± SD (n = 3). *P < 0.05, **P < 0.01 vs. CORT; ###P < 0.001, ***P < 0.001, ****P < 0.0001 vs. control. CTL, control; ART, Artemisinin; CORT, exposed to corticosterone only; ART/CORT, co-treated with artemisinin and corticosterone.

We first examined whether artemisinin could protect PC12 cells from CORT-induced cell toxicity. Figure 1A displays the chemical structure of artemisinin. The results of MTT assay showed that the viability of PC12 cells exposed to 200 μM of CORT for 48 h was significant decreased by 30% (Figure 1B), thus this concentration was chosen for further study. We further co-treated PC12 cells with 200 μM of CORT and different concentrations of artemisinin. The MTT results showed that artemisinin dose-dependently attenuated the loss of cell viability caused by CORT. Specifically, 25 μM of artemisinin restored cell viability to over 95% of that in the control group (Figure 1C). Furthermore, the effect of artemisinin on LDH release in CORT-treated PC12 cells was examined. The results indicated that treating PC12 cells with 200 μM corticosterone for 48 h induced a significant increase in LDH leakage compared to the control group (P < 0.01). Conversely, the addition of artemisinin significantly reduced corticosterone-induced LDH release, as shown in Figure 1D.

### 3.2 Artemisinin decreased intracellular ROS levels and attenuated CORT-induced MMP reduction in PC12 cells

**FIGURE 2:**
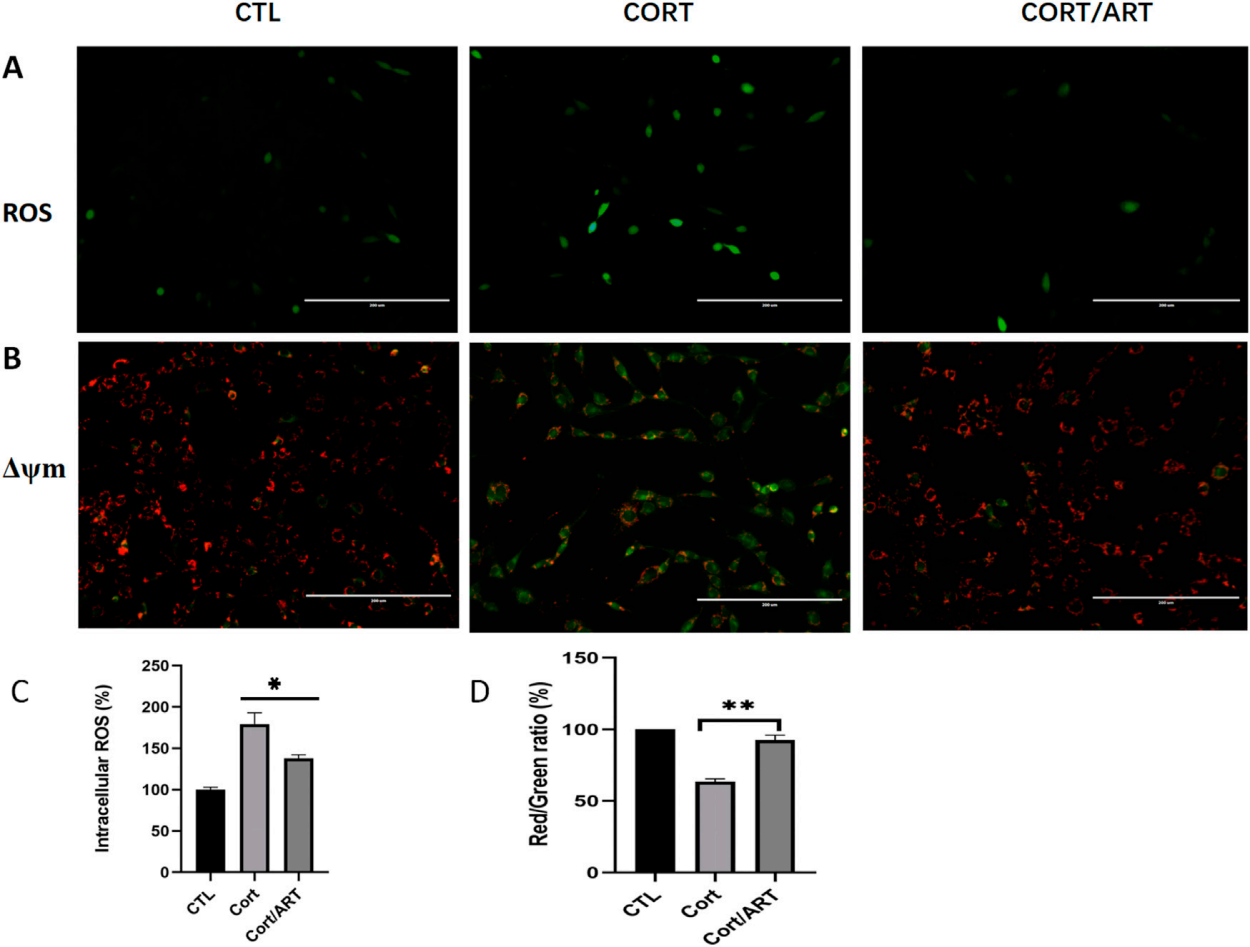
Artemisinin decreased intracellular ROS levels and attenuated CORT-induced ΔΨm reduction in PC12 cells. **(A)** PC12 cells were treated with 25 μM artemisinin or 200 μM CORT for 48 h, followed by fluorescent visualization of ROS production. CORT-treated cells displayed green fluorescence, indicating increased ROS production (n = 3; scale, 200 μm). **(B)** PC12 cells were treated with 25 μM artemisinin or CORT for 48 h, and MMP was analyzed using the JC-1 assay. The decline in membrane potential was indicasted by the shift in fluorescence from red to green, as shown by JC-1 (n = 3; scale, 200 μm). **(C,D)** Quantitative analysis of intracellular ROS in **(A)** and JC-1 in **(B)**, respectively. Data are presented as mean ± SD (n = 3). *P < 0.05, **P < 0.01 versus CORT.

As shown in Figure 2A, exposure of PC12 cells to 200 μM CORT for 48 h resulted in a significant increase in green fluorescent signals, indicating that CORT induced oxidative stress. In contrast, co-treatment with artemisinin (25 μM) for 48 h resulted in a reduction of green fluorescent signals. As shown in Figure 2C, quantitative analysis demonstrates that the intracellular ROS level in CORT-treated PC12 cells significantly increased to 179.5% compared to the control value (100%, P < 0.001). However, co-treatment with 25 μM artemisinin significantly reduced that value to 137% (P < 0.01).

Mitochondria generate membrane potential through the activity of enzymes in the electron transport chain. During apoptosis, the collapse of MMP coincides with the opening of mitochondrial permeability transition pores, leading to the release of cytochrome C into the cytoplasm and triggering downstream events in the apoptotic cascade. To study whether CORT-induced apoptosis is associated with the loss of MMP, we performed JC-1 assay and found that MMP was indeed significantly decreased in PC12 cells after 48 h of treatment with 200 μM CORT, whereas artemisinin reversed this effect (Figures 2B,D). These findings suggest that artemisinin may have a positive impact on mitochondrial function.

### 3.3 Effect of artemisinin on corticosterone-induced apoptosis

**FIGURE 3:**
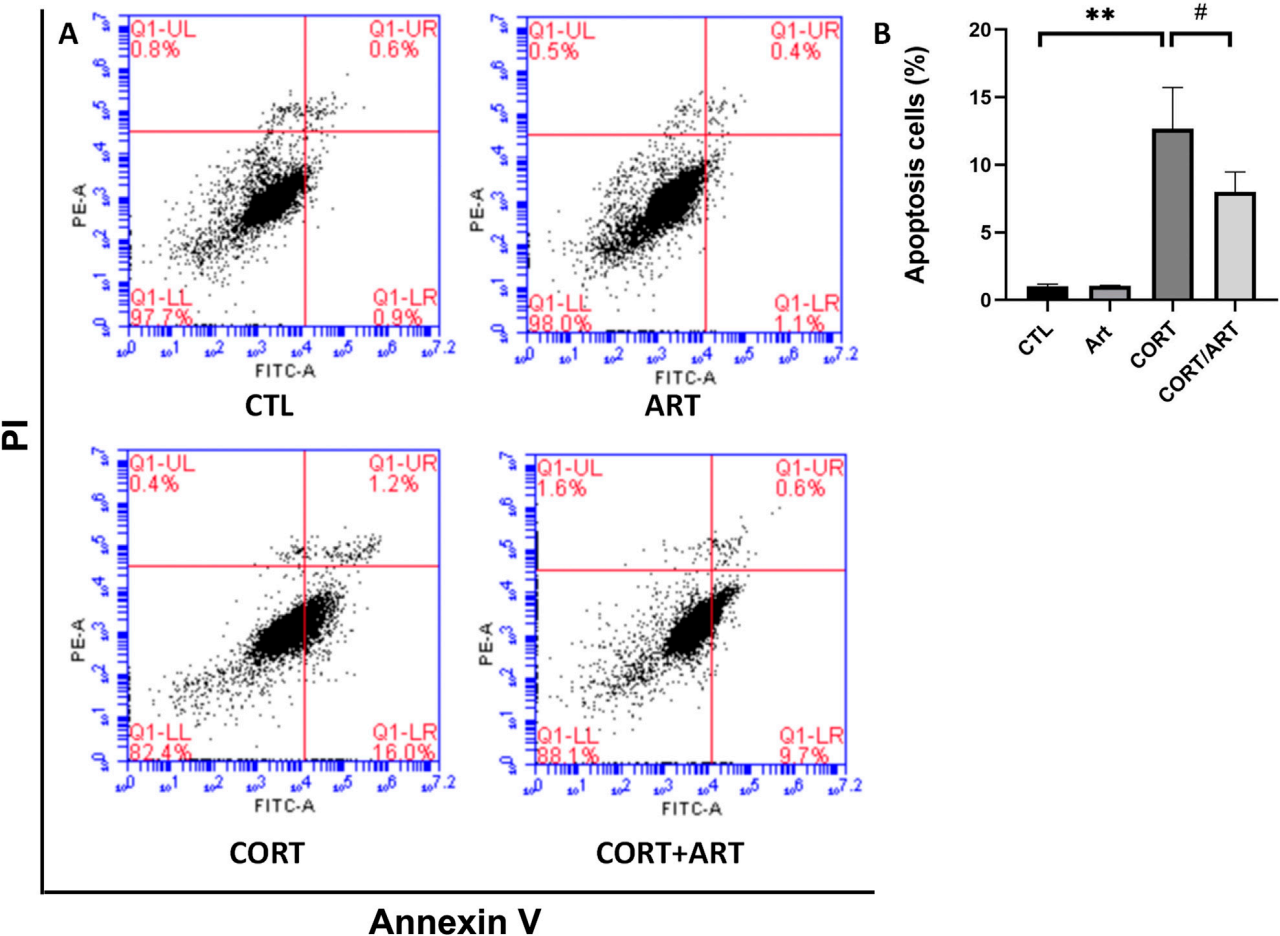
Artemisinin confers cytoprotective effects towards CORT-induced apoptosis in PC12 cells. **(A)** Apoptotic cell death in PC12 cells was analyzed using Annexin V-FITC/PI staining, detected byflow cytometry and quantified as the apoptosis rate **(B)**. Data are presented as mean ± SD (n = 3). **P < 0.01 vs. control; #P < 0.5 vs. CORT.

Annexin V-FITC is a fluorescent probe that binds to phosphatidylserine in the presence of calcium. As shown in Figure 3, treatment with 200 μM CORT for 48 h significantly increased the percentage of Annexin V+/PI + cells, indicating a substantial increase in the apoptosis rate of PC12 cells following CORT-induced damage. However, treatment with 25 μM artemisinin significantly reversed this effect, suggesting that artemisinin can mitigate corticosterone-induced apoptosis in PC12 cells.

### 3.4 Artemisinin confers neuroprotective effect through AKT and ERK signaling pathways

**FIGURE 4:**
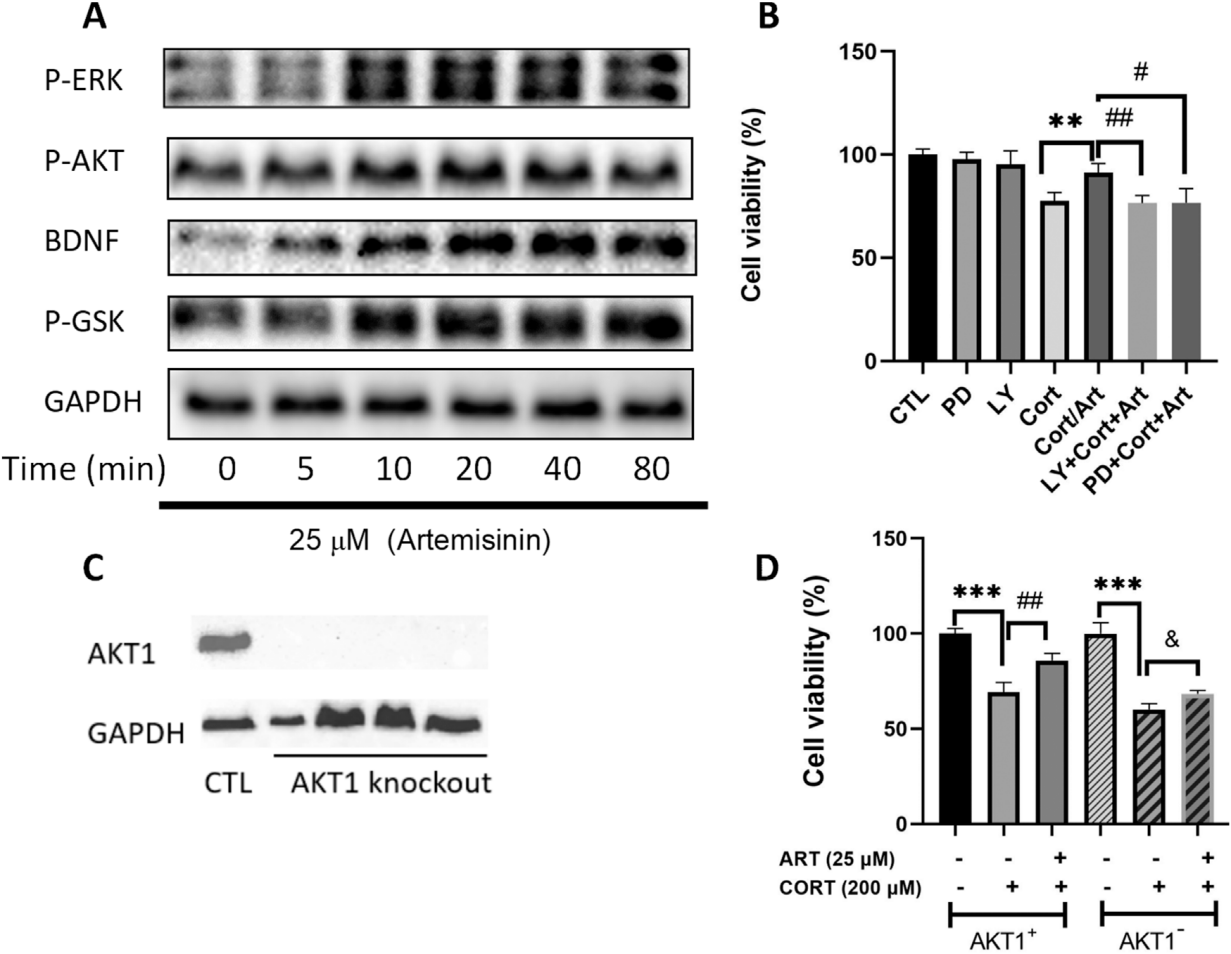
AKT and ERK signaling are involved in the protective effects of artemisinin on PC12 cells against CORT-induced oxidative damage. **(A)** PC12 cells were treated with artemisinin at 25 μM for various durations (0, 5, 10, 20, 40, and 80 min), and the phosphorylation levels of ERK, AKT, GSK, and BDNF were detected by Western blot analysis. **(B)** PC12 cells were preincubated with 25 μM of the PI3K inhibitor (LY294002) and the MEK inhibitor (PD98059) for 30 min, followed by treatment with ART and CORT for 48 h. Cell viability was then assessed using the MTT assay. **(C)** AKT1 knockout cell clones were harvested, and knockout efficiency was confirmed by Western blot analysis. **(D)** Normal and AKT1 knockout cells were treated with ART and CORT for 48 h. Cell viability was determined using the MTT assay. Data are presented as means ± SD (n = 3), *P < 0.05, and P < 0.05, **P < 0.01, ##P < 0.01, ***P < 0.001, ###P < 0.001.

The AKT and ERK signaling pathways have been shown to play crucial roles in promoting cell survival and combating apoptosis. To determine whether artemisinin exerts its anti-apoptotic effects by modulating the AKT and ERK pathways, we treated PC12 cells with varying concentrations of artemisinin for different durations and then extracted proteins to analyze the expression levels of related proteins. We found that artemisinin upregulated the expression of phosphorylated AKT and ERK proteins in a time-dependent manner (Figure 4). Additionally, artemisinin also increased the expression of BDNF and phosphorylated GSK in a time-dependent manner. To further confirm whether the PI3K/AKT and ERK signaling pathways mediate the neuroprotective effect of artemisinin, PC12 cells were pre-treated with the specific PI3K inhibitor LY294002 (25 μM) and the MEK inhibitor PD98059 (25 μM) for 30 min. Subsequently, the cells were exposed to 200 μM CORT in the presence or absence of artemisinin, and cell viability was assessed using the MTT assay. The results (Figure 4B) showed that both inhibitors significantly blocked the cytoprotective effect of artemisinin. Moreover, knocking down AKT1 (Figure 4C) in PC12 cells also declined the protective effect of artemisinin against CORT (Figure 4D). These results further confirm the involvement of the AKT and ERK pathways in the neuroprotective action of artemisinin.

### 3.5 Neuroprotective effects of artemisinin against CORT-induced injury in primary hippocampal neurons

**FIGURE 5:**
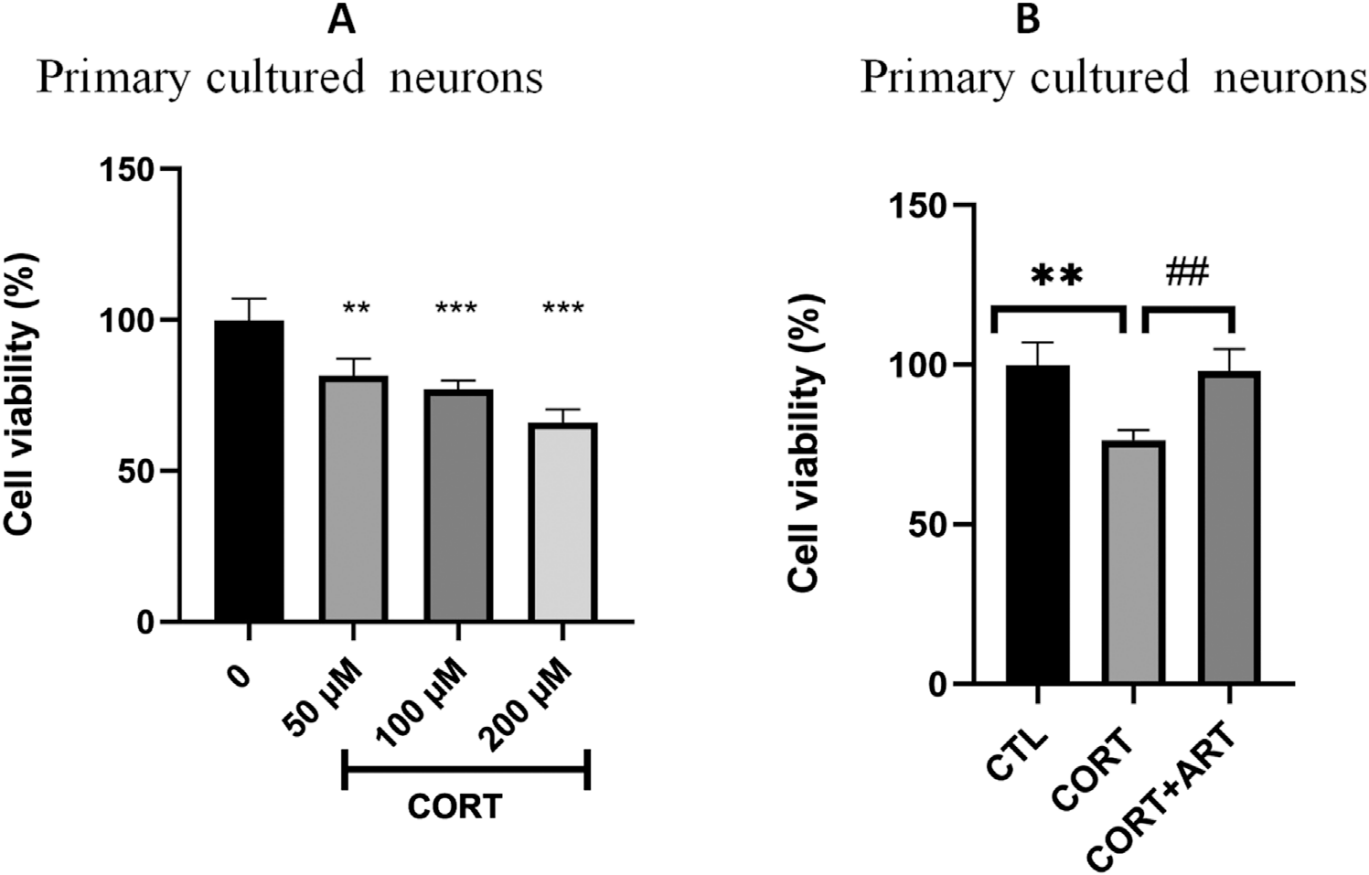
Artemisinin protected primary cultured hippocampal neurons against CORT-induced damage. **(A)** Cytotoxicity of CORT on primary hippocampal neurons. Primary hippocampal neurons were treated with different doses of CORT for 48 h, then cell viability was measured using the MTT assay. **(B)** Primary hippocampal neurons were treated with CORT (100 μM) and artemisinin (25 μM) for 48 h, then cell viability was measured using the MTT assay. The data are represented as the mean ± SD (n = 3). **P < 0.01 and ***P < 0.001 versus control group; ##P < 0.01 versus CORT group.

To investigate if the neuroprotective effect of artemisinin against CORT-induced toxicity is not only limit to PC12 cells line, the neuroprotective effect of artemisinin on primary hippocampal neurons was also examined. The viability of primary cultured neurons was significantly reduced in a dose-dependent manner following treatment with CORT concentrations of 50, 100, and 200 μM (Figure 5A). As shown in Figure 5B, artemisinin (25 μM) was also able to protect hippocampal neurons from the deleterious effects of CORT.

### 3.6 Artemisinin improved depression-like behaviors in CUMS mice

**FIGURE 6:**
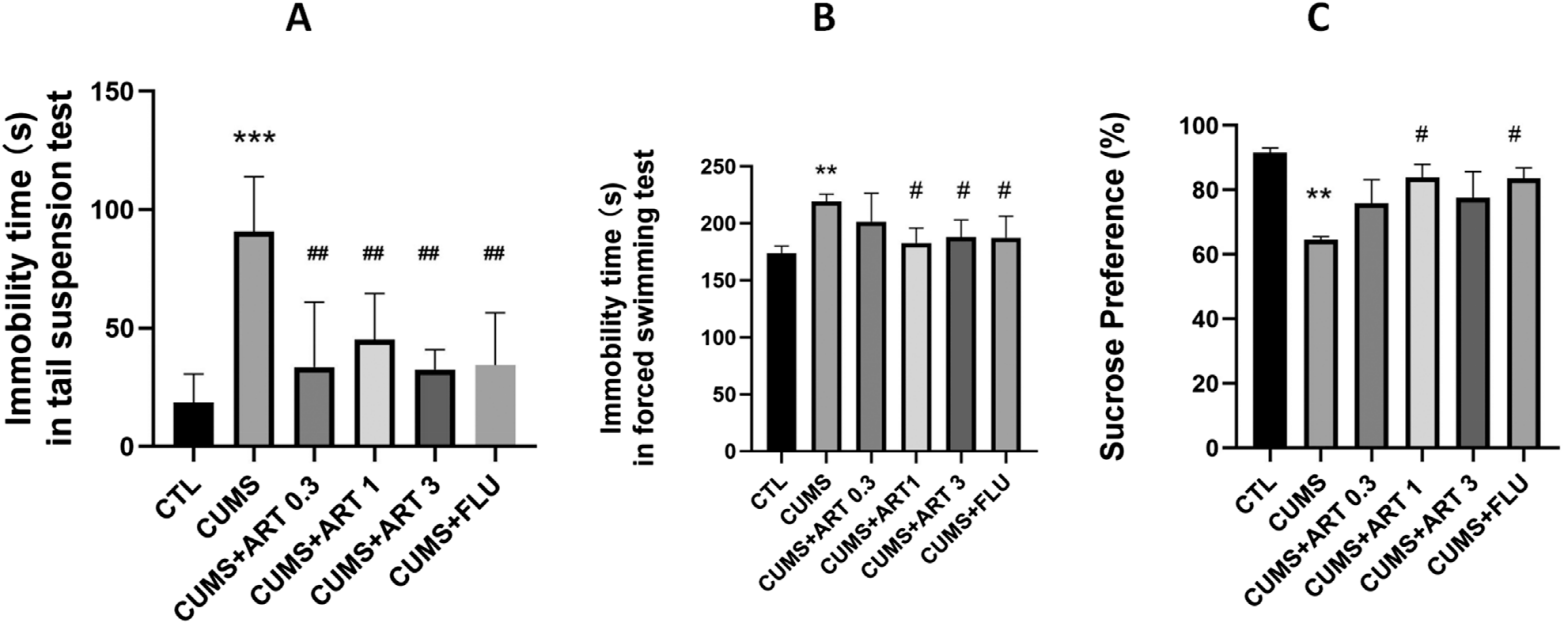
Artemisinin improved depression-like behaviors in CUMS mice. Effect of artemisinin on the immobility duration of mice in the tail suspension test **(A)** and the forced swimming test **(B)**, as well as on sucrose preference **(C)** in stressed mice. The tail suspension test, forced swimming test, and sucrose preference test were carried out 5 weeks after CUMS. Results are presented as mean ± SD (n = 8 per group). **p < 0.01 and ***p < 0.001 versus the control group; #p < 0.05 versus the CUMS group. CUMS: exposed to chronic unpredictable mild stress; ART 0.3: treatment with 0.3 mg/kg dose of artemisinin; ART 1: treatment with 1 mg/kg dose of artemisinin; ART 3: treatment with 3 mg/kg dose of artemisinin; FLU: treatment with 10 mg/kg dose of fluoxetin.

In this study, we evaluated the antidepressant-like activity of artemisinin in the CUMS mice model and used fluoxetine as a reference positive drug. As shown in Figures 6A,B, the immobility duration significantly increased in the CUMS-induced depressive mice in the tail suspension and the forced swimming test. While treatment with artemisinin or fluoxetine can all significantly decreased the immobility duration in both tail suspension and forced swimming tests compared to the CUMS group, and there was no significant difference in the immobility duration between the artemisinin group and the fluoxetine group in the two tests. Similarly, we also assessed depression-like behavior by sucrose preference test in CUMS mice. As shown in Figure. 6C, 5-week CUMS exposure significantly decreased the percentage of sucrose consumption in the CUMS mice as compared to control group; 1 mg/kg dose artemisinin administration significantly and dose-dependently reversed the decrease in sucrose preference in the stressed mice. However, the doses of 0.3 mg/kg and 3 mg/kg artemisinin did not show significant difference in sucrose consumption after 4 weeks of treatment.

### 3.7 Artemisinin stimulated the activation of AKT/GSK/NRF2/HO1 and BDNF/TrkB/ERK/CREB the signaling pathway in mice brain

**FIGURE 7:**
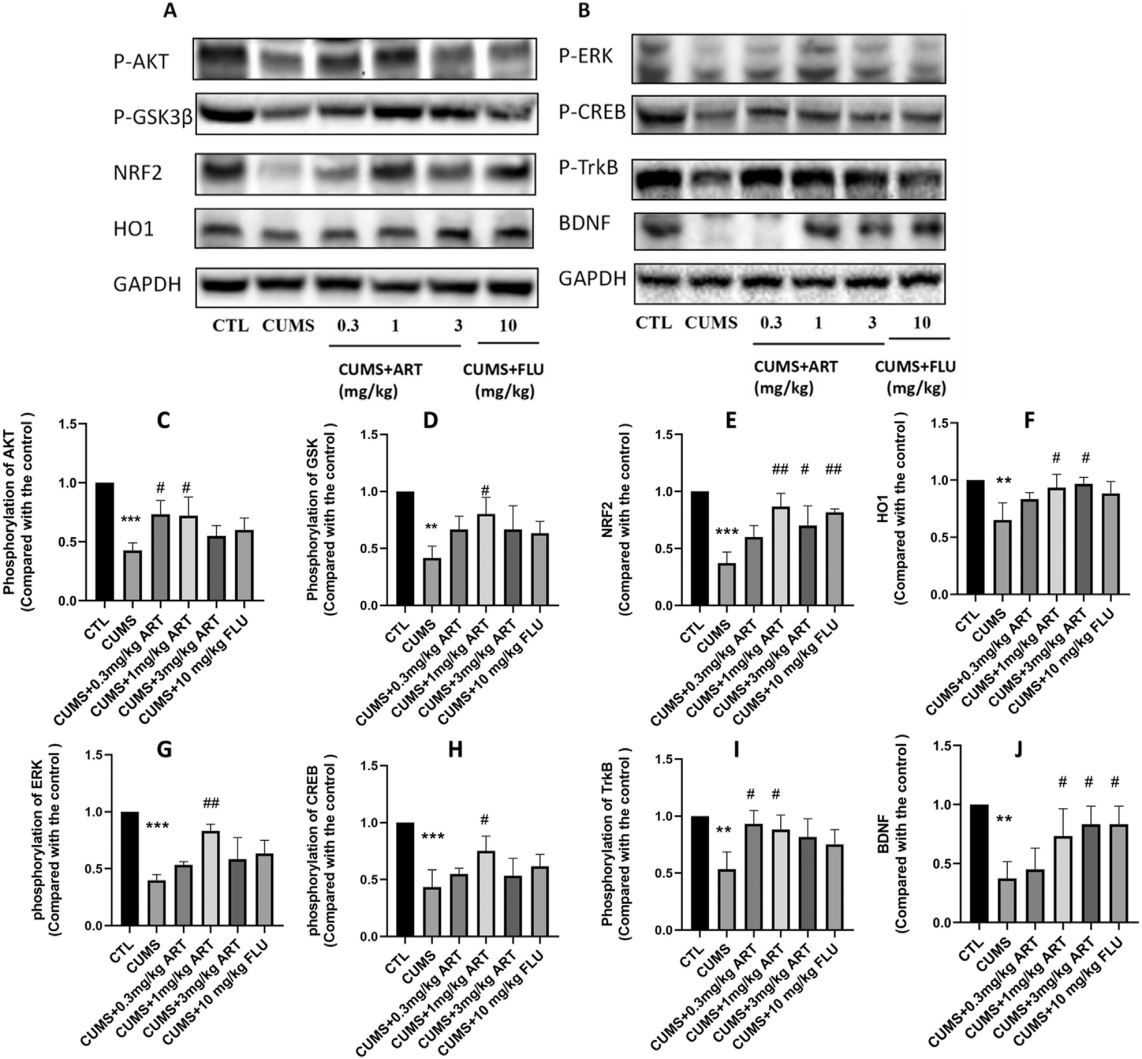
Mechanism of the effect of artemisinin on depression. Mice were treated with different doses of artemisinin (0.3 mg/kg, 1 mg/kg, and 3 mg/kg) once daily for 4 weeks. Hippocampal protein was analyzed by Western blotting. **(A)** Protein expression levels of NRF2, HO1, and phosphorylation of AKT/GSK3β (Ser9). **(B)** Protein expression levels of BDNF and phosphorylation of ERK, CREB and TrkB. **(C–F)** Quantitative analysis of **(A)**. **(G–J)** Quantitative analysis of **(B)**. Results are shown as the mean ± SD of (n = 3). **P < 0.01 and ***P < 0.001versus control group; #p < 0.05, ##p < 0.01, versus CORT group.

To examine whether Artemisinin confers anti-depression effect via AKT/GSK/NRF2/HO1 and BDNF/TrkB/ERK/CREB pathway, we performed Western blot analysis to check the levels of phosphorylation of those proteins (Figure 7A). The results indicated that artemisinin treatment increased the level of AKT, GSK, ERK and CREB phosphorylation, as well as stimulated the expression of NRF2, HO1 and BDNF in brain extracts of CUMS mice (Figure 7B). Quantification results of the protein bands from the western blots are presented in Figures 7C–J.

### 3.8 Artemisinin mitigates depression-associated glial dysregulation and neurogenesis impairment in CUMS model

**FIGURE 8:**
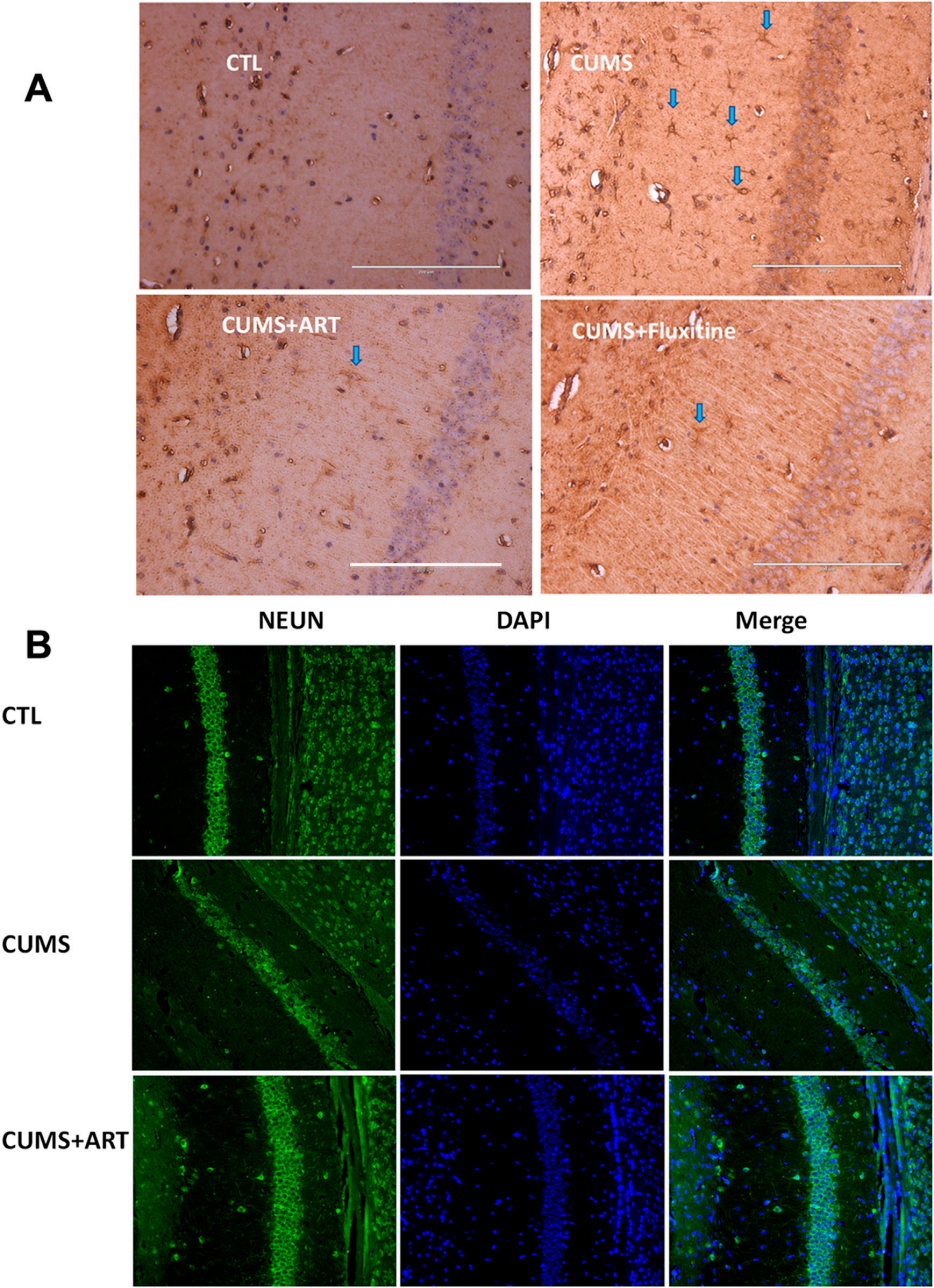
Artemisinin modulates astrocyte activation and neuronal maturation in the hippocampal CA1 region of CUMS mice. **(A)** Representative immunohistochemical images (200×) of GFAP in the hippocampal CA1 region. Blue arrows highlight GFAP^+^ cells. Fluoxetine treatment serves as the positive control. Scale bars = 200 μm (white lines on the pictures) **(B)** Representative immunofuorescence images for NeuN (green) and DAPI (blue) in the mice hippocampal CA1 region.

CUMS induces pathological alterations in hippocampal astrocytes and neurogenesis, both hallmarks of depression. To evaluate artemisinin’s cellular effects in this context, we analyzed astrocyte activity and neuronal maturation in the CA1 region, with fluoxetine serving as a positive control. Immunohistochemical staining for GFAP, a marker of astrocyte activation, revealed a significant increase in GFAP + cells in CUMS mice compared to controls, reflecting stress-induced astrocytic hyperactivity. Artemisinin treatment markedly reduced GFAP levels, similar to the effect of fluoxetine, suggesting its potential to normalize astrocyte activity in the depressed hippocampus (Figure 8A). Concurrently, we assessed neurogenesis by quantifying NeuN + mature neurons, which are critical for functional hippocampal circuitry. CUMS exposure caused a severe loss of NeuN + neurons, consistent with impaired neurogenesis in depression. Artemisinin administration significantly restored NeuN^+^ cell numbers, indicating its capacity to counteract stress-induced neuronal deficits (Figure 8B). These results collectively highlight artemisinin’s dual role in ameliorating depression-associated glial dysregulation and neurogenesis impairment.

## 4 Discussion

Although significant efforts have been made to improve the diagnosis and treatment of depression, a large percentage of patients still do not respond well to current interventions that modulate the monoaminergic system. Additionally, these drugs may be accompanied by undesirable side effects. Therefore, there is a critical need for new therapeutic drugs with high efficacy and low toxicity. In this study, we demonstrated that artemisinin significantly increased the viability of PC12 cells and neurons treated with CORT *in vitro*. It markedly inhibited CORT-induced leakage of LDH, production of ROS, and dysfunction of MMP in PC12 cells. *In vivo*, chronic administration of artemisinin improved CUMS-induced depression-like behaviors in both the FST and the TST, which are two commonly used screening tests for antidepressant-like activity (Castagné et al., 2010). Our study presents novel findings on the neuroprotective and antidepressant-like effects of artemisinin in both *in vitro* and *in vivo* models, indicating its potential as a drug treating depression.

Our findings that artemisinin reverses CORT-induced apoptosis and mitochondrial dysfunction extend prior evidence of its neuroprotective capacity. This discovery is supported by earlier studies which, for example, demonstrated that artemisinin can stimulate neuronal cell viability and protect against certain types of cellular stress in neuronal-like cells (Pukhov et al., 2025; Zhao et al., 2019a; Zhao X. et al., 2020; Zheng et al., 2016). While these effects are not directly linked to antidepressant outcomes, they support the possibility that artemisinin may enhance brain health and resilience.

Our data demonstrate that artemisinin effectively attenuated corticosterone-induced ROS overproduction in neuronal-like cell models, which appears to contrast with its well-established role as a pro-oxidant antimalarial agent. This apparent paradox may be explained by the context-dependent redox activity of artemisinin. Its antimalarial action is mediated by iron-dependent cleavage of the endoperoxide bridge—primarily by heme iron derived from hemoglobin digestion within the parasite—leading to localized radical formation and cytotoxic effects specifically targeting Plasmodium. In non-malarial systems, artemisinin and its derivatives have been reported to exhibit antioxidant properties, possibly through free radical scavenging or modulation of cellular antioxidant pathways. Thus, the compound’s redox behavior may shift from pro-oxidant in parasite-infected erythrocytes to antioxidant in neuronal cells, reflecting differences in local chemical microenvironments and metal availability (Posadino et al., 2023; Zheng et al., 2024).

Notably, the result show that after 4 weeks of treatment, only the 1 mg/kg ART group produced a significant reversal of the CUMS-induced decrease in sucrose preference; the 0.3 mg/kg and 3 mg/kg groups did not reach significance, reflecting an inverted-U dose–response, which has been frequently reported in neuropharmacological studies (Baldi and Bucherelli, 2005; Stone et al., 2011).

Previous studies have primarily focused on artemisinin’s anti-inflammatory and anti-cancer properties. Although depression is associated with neuroinflammation, limited investigation has been conducted into artemisinin’s potential antidepressant effects. A study found that dihydroartemisinin, a derivate of artemisinin, improved performance of mice in open-field test and closed-field test, implies that dihydroartemisinin can improve depression-like behavior (Gao et al., 2020). A recent study also reported that dihydroartemisinin protected mice from CUMS-induced depression-like behaviors, evidenced by sugar water preference, forced swimming and tail suspension experiments, and the effect is associated with gut microbes (Tang et al., 2024). However, whether artemisinin has antidepressant effect was unclear. Our study fills this gap by demonstrating that artemisinin can mitigate CORT-induced neuronal damage and improve depressive symptoms in CUMS mice model, highlighting its potential as a novel therapeutic agent for depression. Furthermore, emerging evidence suggests that the antidepressant-like effects of artemisinin derivatives may be enhanced by interaction with γ-aminobutyric acid (GABA). Hybrid compounds of dihydroartemisinin and GABA have demonstrated significant antidepressant-like effects in cell models, further supporting the therapeutic potential of this pathway (He et al., 2021). Whether artemisinin confers its antidepressant effect by mediating gut microbes as dihydroartemisinin does or through GABAergic mechanisms suggested by these hybrid studies, are compelling questions requiring further investigation.

Our study also provides mechanistic insights into the neuroprotective and antidepressant effects of artemisinin. We found that artemisinin activates AKT and ERK pathways in PC12 cells, and results of both pharmacological inhibition and genetic knocking down reveal that its neuroprotective effects are dependent on these pathways. Our findings are consistent with existing literature on the role of AKT and ERK signaling in neuroprotection and neurological functions. Studies have shown that activation of the AKT pathway promotes neuronal survival and growth (Dudek et al., 1997), while ERK signaling is essential for synaptic plasticity and cognitive functions (Thomas and Huganir, 2004). However, our study links these pathways to the neuroprotective effects of artemisinin, thereby expanding the potential applications of this compound beyond its traditional uses.

A recent work studied pathogenesis of depression in four stress-induced models (Li et al., 2023). By combining proteomic and metabolomic approaches, they found that molecular alterations in depression converge on a common AKT and ERK molecular pathways. Indeed, the antidepressant effects of many interventions are associated with these two pathways. For example, creatine and taurine mixtures showed antidepressant effects by mediating Akt and ERK/BDNF pathways (Kim et al., 2020). The anti-depressant effect of vanillic acid was Akt-dependent, although was ERK-independent (Chuang et al., 2020). The AKT pathway was upregulated by dihydroartemisinin, although this was not further validated by using AKT inhibitors or knockdown experiments. Our mechanistic study also in agreement with abovementioned studies, highlighting the essential role of AKT and ERK pathways in the pathogenesis of depression. Further studies of these pathways could contribute to developing pharmacological interventions of depression.

Glia cells play a key role in brain inflammation and depression (Novakovic et al., 2023; Wang et al., 2022; Zhou et al., 2021). Previous study has revealed that artemisinin could alleviate sepsis-associated neuroinflammation and cognitive impairment by modulating the activity of microglia (Lin et al., 2021). In the present study, we found that artemisinin inhibited the activity of astrocyte in CUMS mice, presenting a more comprehensive role of artemisinin in modulating glia cells and neurological disorders. Depression is also characterized by impaired neurogenesis in the brain (Hanson et al., 2011). We also found that artemisinin promoted neurogenesis in the hippocampal CA1 of CUMS mice. These findings highlight the cellular mechanisms underlying its antidepressant effects.

## 5 Conclusion

In conclusion, our study highlights the potential of artemisinin as a novel therapeutic agent for depression, providing robust evidence of its neuroprotective and antidepressant-like effects in both cellular and animal models. By modulating key signaling pathways, artemisinin offers a promising alternative to traditional antidepressants with the potential for lower toxicity. Future research should aim to translate these findings into clinical settings, paving the way for new treatments for depression.

## Data Availability

The original contributions presented in the study are included in the article/supplementary material, further inquiries can be directed to the corresponding author.

## Glossary

AD: Alzheimer’s Disease
AKT: Protein Kinase B
ART: Artemisinin
BBB: Blood-Brain Barrier
BCA: Bicinchoninic Acid
BSA: Bovine Serum Albumin
CORT: Corticosterone
CST: Cell Signaling Technology
CUMS: Chronic Unpredictable Mild Stress
DAPI: 4′,6-diamidino-2-phenylindole
DMEM: Dulbecco’s Modified Eagle’s Medium
DMSO: Dimethyl sulfoxide
ERK: Extracellular Signal-Regulated Kinase
FDA: Food and Drug Administration
FBS: Fetal Bovine Serum
FST: Forced Swim Test
H&E: Hematoxylin and Eosin
HPA: Hypothalamic-Pituitary-Adrenal
HRP: Horseradish Peroxidase
LDH: Lactate Dehydrogenase
MAOIs: Monoamine Oxidase Inhibitors
MEK: Mitogen-Activated Protein Kinase/Extracellular Signal-Regulated Kinase Kinase
MMP: Mitochondrial Membrane Potential
PC12: Pheochromocytoma cell line
PD: Parkinson’s Disease
PI3K: Phosphoinositide 3-Kinase
ROS: Reactive Oxygen Species
SPT: Sucrose Preference Test
SSRIs: Selective Serotonin Reuptake Inhibitors
TCAs: Tricyclic Antidepressants
TST: Tail Suspension Test
